# Swine industry perspectives on the future of pig farming

**DOI:** 10.1017/awf.2024.2

**Published:** 2024-02-13

**Authors:** Marina AG von Keyserlingk, Jillian Hendricks, Beth Ventura, Daniel M Weary

**Affiliations:** 1 Animal Welfare Program, Faculty of Land and Food Systems, The University of British Columbia, 2357 Main Mall, Vancouver, BC, Canada V6T 1Z4; 2 Department of Life Sciences, University of Lincoln, Lincoln, Lincs LN6 7DL, UK

**Keywords:** animal welfare, employee training, focus groups, qualitative methods, social licence, sustainability

## Abstract

Understanding the views of those working along the value chain reliant on livestock is an important step in supporting the transition towards more sustainable farming systems. We recruited 31 delegates attending the Pig Welfare Symposium held in the United States to participate in one of six focus group discussions on the future of pig farming. Each of these six group discussions was subjected to a thematic analysis that identified four themes: (1) technical changes on the farm; (2) farm and industry culture; (3) the farm-public interface; and (4) sustainability. The results of this study illustrate the complexity and diversity of views of those working along the associated value chain within the swine industry. Participants spent the majority of their time discussing current challenges, including technical challenges on the farm and public perception of pig farms. Participants were more hesitant to discuss future issues, but did engage on the broader issue of sustainability, focusing upon economic and environmental aspects.

## Introduction

Increasingly, various sectors of society (Dockès & Kling-Eveillard 2006; Benard & de Cock Buning 2013; Vandresen & Hotzel 2021a,b) are calling into question the practice of intensive pig production systems, likely due in large part to the reliance on systems that severely restrict animal movement. These concerns have resulted in legislative requirements concerning how pigs are housed (Broom 2017), including the banning of gestation stalls within the European Union (EU) more than a decade ago (EU Directive 2008/120/EC 2013). The push for such changes has often come from those outside of the sector, and farmers and others affiliated with the industry are sometimes left defending practices, arguing that criticisms of the sector are rooted in public ignorance of farm practices (e.g. Benard & de Cock Buning 2013).

In the United States, pig farming is largely self-regulated, with few laws governing how animals are cared for on-farm. The federal Animal Welfare Act (1966) does not govern on-farm practice, and relatively few US states have legislation about pig housing (Ufer 2022). The ability of an industry to self-regulate is associated with the granting of social licence, which is provided by the public with the expectation that industry practices will conform to the values held by the rest of society (see Gunningham *et al.* 2004; Rollin 2011). However, societal values are in flux, so organisations allowed to self-regulate must stay in sync with values to retain their licence to practice (for further discussion, see Hampton *et al.* 2020).

To align with societal values first requires that they are well-understood, as informed by research on public perspectives on farming practices. For instance, Sato *et al.* (2017) surveyed primarily millennial US participants, inviting them to respond to the open-ended question: “What do you consider to be an ideal pork/pig farm and why are these characteristics important to you?” Many participants responded that pigs should be given sufficient space and were concerned about housing that restricts the movement of pigs, a finding also echoed in other studies (Ryan *et al.* 2015; Yunes *et al.* 2018). Vandresen and Hötzel (2021a,b) found that Brazilian citizens viewed farrowing crates that restrict the movement of sows to be cruel and unnatural, and instead favoured outdoor housing systems that were perceived to allow a more natural life.

In addition to understanding the perspectives of those outside of the industry, it is important to understand the views of those from within, including both farmers, nutritionists and others working in positions along the value chain (e.g. veterinarians and academics/scientists) that depend on the production of pigs, in part because these individuals are well suited to provide leadership or to surmount barriers to meaningful change (Jagosh *et al.* 2012). Industry-led changes may help avoid future disruptions, including the imposition of legislated changes (Ceccato *et al.* 2022), that would otherwise be disruptive to those working in the industry. The aim of the current study was therefore to assess the views of people, including farmers and others with professional involvement in the pig industry, as to the desired characteristics of their industry.

## Materials and methods

This study took place at the 2nd annual Pig Welfare Symposium, hosted by the United States National Pork Board and held in Minneapolis, MN, United States, November 13–14, 2019. The symposium was designed to be a forum for sharing ideas and fostering dialogue about animal welfare among stakeholders in the pig industry, and recruited animal welfare scientists, pig producers and care staff, and industry affiliates amongst their delegates.

### Ethical approval

This study was approved by the ethics board of The University of British Columbia (H18-02880).

### Positionality statement

Researchers’ world views shape their research at every stage, and while not a panacea, naming how one’s past experiences may intersect with their research is intended to improve transparency of the research process (Holmes 2020). MvK and DMW are Professors in the UBC Animal Welfare Program who have collaborated for over two decades, and each has published work on public attitudes to pig production. Prior to their appointments at UBC, DMW worked as a researcher for Agriculture and Agri-Food Canada, focusing on issues relating to swine welfare, and MvK worked for the animal feed industry, working directly with livestock farmers, including pig farmers. JH completed her undergraduate in Applied Biology at UBC and is currently a PhD student at the University of Bristol, UK. She has no previous experience in the pig industry but has published a series of articles that involved qualitative analyses of interviews and focus groups. BV completed her PhD in the UBC Animal Welfare Program and engages in livestock welfare and social science research, including veterinary student attitudes to pig welfare.

### Data collection

We sought to conduct these focus groups with symposium participants in part as an engagement mechanism to facilitate inter- and intra-stakeholder discussions about desired futures for the swine industry. While we hoped to ensure a high level of participation by those directly involved in pig farming, participation in the focus groups was open to any symposium attendee. Attendees were made aware of the possibility of participating in focus groups through announcements made during the symposium and through signage that was placed outside of the room where the focus groups took place. Ultimately, a convenience sample of 31 symposium participants (~15% of symposium registrants) was recruited, and randomly assigned to one of six focus groups (on average each focus group included 4–6 participants). Participants worked in a number of other positions within the industry (n = 12; farmers, nutritionists, geneticists) or stated that they were academics/scientists (n = 12), veterinarians (n = 6), or worked in the not-for-profit sector (n = 1). Focus group sessions were based upon a semi-structured question guide (Table 1), and were moderated by four facilitators (MvK, BV, DMW and one volunteer graduate student from the University of Minnesota); all four had previous experience facilitating focus groups on a variety of animal welfare topics. The four facilitators met before the focus group sessions to review the interview guide.

**Table 1.** Semi-structured question guide used for six focus groups of 31 volunteer participants attending a conference focused on pig welfare, where they were asked discuss their views on the future of pig farming

Semi-structured discussion guide
Our context: Let’s fast forward to the year 2050. You’re hoping that your grandchildren will take over the farm. In your ideal world, what does this farm look like? What characteristics would you consider to be ‘must haves’ in order for them to want to take over the farm?
Describe in words, the characteristics of this farm, or feel free to draw a picture. Write down on the ‘sticky-notes’, your top 3–5 ‘must-haves’ for this farm
PROBE: Tell me more about the farm itself- what does it look like? the animals, the facility itself, the working environment
Feel free to share why these characteristics are important to you. Are there any farm characteristics that are common today that you think would make farming less attractive for your grandchildren? Write down on sticky notes, your top 3–5 ‘must-have nots’ for this farm
PROBES: Go around the table- anyone willing to share what you came up with? Does everyone agree? Any different perspectives?
Let’s return to the present: what do we need to do as an industry to help achieve that farm? Again, please start by writing down a few specific examples.
PROBES: Go around the table- anyone willing to share what you came up with? Does everyone agree? Any different perspectives?
That concludes the focus group. Thank you again for your participation.

Each focus group began with a brief introduction by the facilitator to describe the aim of the session. Participants were then asked to read and sign the consent form, explaining that the results of the study would be prepared for submission to a peer-reviewed publication. All were provided the option of not signing and instead joining another focus group where the discussions would not be recorded or used in our analysis, but no one chose this option, and all signed the consent form. To begin, participants were asked to state their pre-assigned anonymous identification (ID) number and to broadly describe their role within the pig industry. This was done to aid the transcription service in assigning text to specific individuals within the focus group.

To facilitate discussion, participants were asked to write 3–5 key words or phrases on notes reflecting what they considered to be ‘must-haves’ relating to pig care on farms 20–30 years into the future. This ‘post-it notes’ approach provided a starting point for discussion and helped jog memory as the discussion progressed (Kontio *et al.* 2004). The note exercise was repeated twice more, first for participants to record their thoughts about which industry practices should be changed (the ‘leave behinds’), and second how they would implement their vision for the future pig industry. Our semi-structured questions were used to ask participants to expand on the words provided in the notes; the question guide included primary questions used by facilitators to initiate discussion on a topic, and secondary questions used as prompts if necessary. Focus groups lasted between 42 and 59 min. Table 2 describes the participants’ employment demographics based on their responses during the introductory phase of the focus groups.

**Table 2.** Participant demographics from six focus groups with 31 representatives of the pig industry (based on participants’ self-reported employment status during the introduction phase of the focus groups); participants were all attending a conference focusing on pig welfare. ‘Industry’ denotes those directly employed in farming, i.e. pig producers and caretakers. The assignment of individuals to the tables (one table per focus group) was done haphazardly as the organisers did not ask the participants their role within the pig industry until after the focus groups had been formed

Profession	n
Industry (i.e. farmers, nutritionists, geneticists)	12
Academic/Scientist	12
Veterinarian	6
Non-profit organisation	1

### Analysis

Audio-recordings were transcribed *verbatim* by a professional transcription service and transcripts checked for accuracy. The second author (JH) coded the transcripts using descriptive coding (Sandowski 2000). Descriptive coding aims to provide a comprehensive summary of events and codes are derived directly from the data (Sandowski 2000). JH began by reading each transcript line-by-line and assigning descriptors to pieces of data that were relevant to the research question. Descriptors were grouped by similarity to create a list of codes and sub-codes, and then codes were clustered into themes, resulting in an organised codebook. The codebook was validated through one round of inter-coder reliability assessment using another trained individual in qualitative analyses who independently coded two transcripts using the codebook. The two coders then met to discuss differences in coding and codebook interpretation, and adjustments to the codebook were made accordingly. JH then coded all of the transcripts using the finalised version of the codebook. All coding was carried out using NVivo (QSR International Pty Ltd, version 12; https://www.qsrinternational.com/nvivo-qualitative-data-analysis-software/home). Despite the use of open-ended questions to prompt the participants to focus on the ‘must haves’, then the ‘leave behinds’ and lastly on how to implement their vision, the primary themes that arose from the conversations were heavily weighted towards solving current issues faced by the industry. We thus elected to not delineate the coding by question; rather, the results were organised into themes and subthemes which included responses to all of the questions posed during the discussions.

Unique identifiers including the participants’ anonymous ID number and focus group ID (e.g. P2F1) are used to report the results below. We also explicitly identify whether the participant worked as a veterinarian, an academic or scientist, and in another position within the industry (i.e. farmer, nutritionist, geneticist). Quotes were selected for inclusion to represent key ideas that emerged from the focus groups. Square brackets (i.e. […]) were used to indicate when a quote was shortened or when we inserted explanatory information to ensure the meaning was maintained. We emphasise the diversity of themes described by participants as opposed to the quantity.

### Data and model availability statement

At the time of the interviews, we obtained consent to subject the transcripts to thematic analyses as part of the process to preparing publication but we did not specifically ask if the anonymised transcripts could be made public. For this reason the raw transcripts are not available.

## Results and Discussion

**Figure 1.**
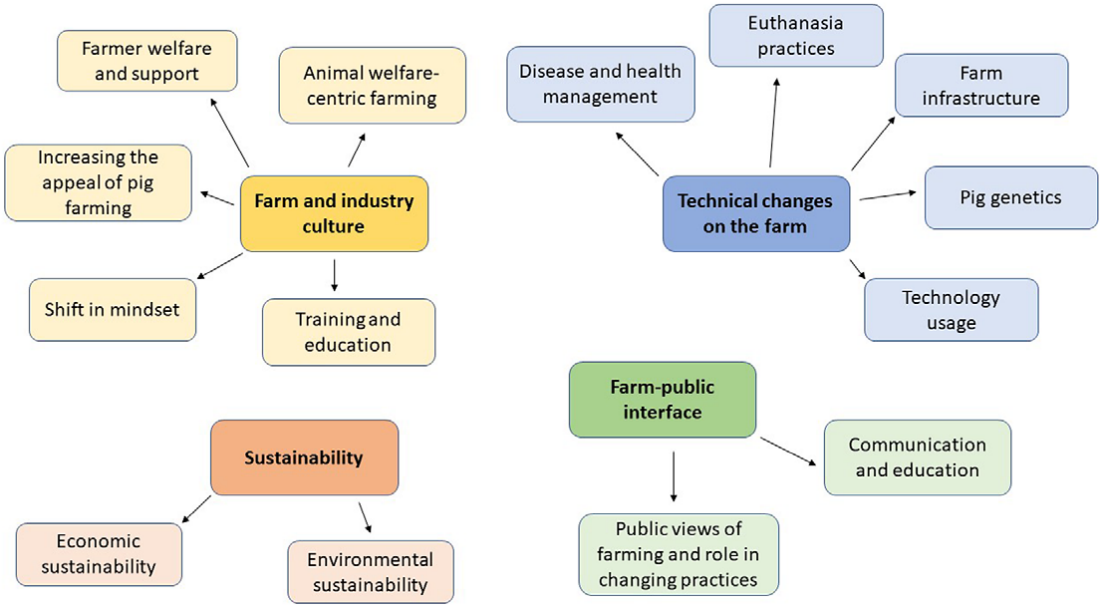
Thematic map of themes and subthemes from focus groups (n = 6) with 31 representatives of the pig industry regarding their views on the future of pig farming. Each colour represents a different theme; farm and industry culture; technical changes on the farm: sustainability; and farm-public interface. Overarching themes are depicted in darker colour and in bold, while subthemes are depicted in a lighter colour.

We identified the following four primary themes: (1) technical changes on-farm; (2) farm and industry culture; (3) farm-public interface; and (4) sustainability. Each of these themes included two to five subthemes (see Figure 1).

### Technical changes on-farm

Participants discussed a variety of management practices needing change on pig farms, including disease and health management, euthanasia practices, farm infrastructure, pig genetics, and technology usage.

#### Disease and health management

Participants discussed the need for better prevention of disease outbreaks on pig farms, for example: “[…] We [need to] keep these viruses from running rampant […] influenza and whatever’s next. You know, it’s not so much the ones that are here, it’s what’s next” [P1F6; veterinarian]. That the participants in this study focused on disease and health management was not unexpected given that previous work has found that those working directly within livestock industries tend to focus on issues relating to animal health and productivity (Te Velde *et al.* 2002; Vanhonacker *et al.* 2008). This emphasis on health is likely driven in part by the desire to improve farm efficiency (Neimi *et al*. 2016, 2017).

Globally there is considerable discussion on reducing dependency on antibiotics in animal agriculture systems (WHO 2022). This topic was also brought up by focus group participants, who discussed the possibility of using alternative methods to prevent disease: P2F6 [veterinarian]: “I think by 2050… everything will be preventative. Vaccinations, organic. Oreganos, apple cider vinegars…. I don’t think we’ll be using antibiotics. So, [we will need] to prevent diseases rather than treat.” Another participant offered a vision of disease-free farms where the need for certain treatments was eliminated: “Disease-free… [no need to] use antibiotics and vaccines [and] no injections,” [P3F1; veterinarian]. A recent Danish study reported that nearly half of consumers are interested in a substantial reduction in antibiotic use in pig production (Denver *et al.* 2021). This view resonates with the growing concern regarding antibiotic resistance in pig farming in several countries, including Australia (Abraham *et al.* 2017) and China (Yang Hong *et al.* 2019).

#### Euthanasia practices

Participants discussed the importance of improving euthanasia practices on farms. As participant P3F1 [veterinarian] described: “[we need a] more effective method of euthanasia on the farms other than CO_2_, or blunt force trauma.” Some work has been conducted on assessing alternative on-farm euthanasia methods in pigs, including captive-bolt guns (Kramer *et al.* 2021) and electrocution (Husheer *et al.* 2020). When assessing swine caretaker characteristics and attitudes toward timely euthanasia, Campler *et al.* (2018) reported that participant attitudes were related to caretaker training and experience, highlighting that approximately 20% (of 84 participants) were “not confident” and lacked sufficient experience to make decisions regarding euthanasia. Training is known to be crucial for delivery of humane euthanasia (McGee *et al.* 2016).

Another participant commented on how end-of-life decisions affect public trust: “In my ideal world, the caretakers and people working boots on the ground are [making good decisions] related to animal care and timely euthanasia, … hopefully [this will] eliminate mistrust in agriculture” [P6, F1 academic/scientist]. Incidents along the value chain that are perceived as negative can erode public trust in the food supply (Sarpong 2014), with issues relating to food animals being particularly salient in reducing trust (Mazzocchi *et al.* 2008).

#### Farm infrastructure

Participants discussed changing several aspects of farm infrastructure on pig farms. For instance, some features of pig facility design were viewed as problematic, although this discussion focused primarily on issues such as longevity of infrastructure, air quality and worker welfare. In the words of one participant: “What’s the cheapest way to get this building up? … What’s going to be the building that’s going to last the longest and benefit me the most?” [P8F2; industry]. Improving air quality in barns was also brought up, for example: “…. you have the higher ceilings, a little bit [lighter], whether it’s skylights or more windows, to make it a better environment for barn workers” [P4F4; academic/scientist].

All six focus groups brought up issues relating to management and housing systems, particularly in relation to restriction of animal movement, an issue of concern for others (e.g. citizens, organic and conventional farmers, veterinarians and pig husbandry advisors: Bergstra *et al.* 2017; public: Yunes *et al.* 2018; Vandresen & Hötzel 2021a,b). Participants discussed changes to pig housing systems, such as transitioning to group housing, and how this transition could be accomplished with minimal disruption:

“I’m interested in seeing where we are going with transitioning from gestation to group housing, and then by 2050, what that means, also, for farrowing. If there’s interest in free farrowing systems or pen systems, group systems, what kind of implementation that would look like and how we could do that in a way that is looking at all aspects of animal health as well as freedom of behaviour” [P3F6; academic/scientist].

The recognition that some facility designs may be problematic was not surprising given the criticisms of restriction of movement associated with gestation stalls (see Ryan *et al.* 2015; Clark *et al.* 2017) and the 2013 legislation in the EU severely curtailing the use of gestation crates (EU Directive 2008/120/EC 2013). For any system to be sustainable it must also be viewed as acceptable by everyone along the value chain, including consumers who purchase pork (Vandresen & Hötzel 2021a,b). It is thus important to understand all stakeholders’ views, including the public, prior to implementing widespread changes in farm management practices (Weary *et al.* 2016).

#### Pig genetics

Participants discussed changes in pig genetics as part of the future of pig farming. One participant discussed this topic in relation to piglet mortality: “I’d like to see a genetic change by 2050 that [results in] litters that can actually survive” [P3F6; academic/scientist]. In previous work on the public acceptance of farrowing crates, participants believed that the genetic selection of sows to produce more piglets was associated with piglet crushing (Vandresen & Hötzel 2021a). Taken together with our findings, there appears to be some sentiment from stakeholders that it is not the housing system *per se* that the public finds to be problematic but rather the ‘system’ and how the animals are shaped to fit in it.

Some participants felt “there’s more that can be done with gene editing with disease prevention” [P3F4; industry]. Whether gene editing is embraced by society remains to be seen, but there is evidence that this technology is less likely to be accepted if viewed primarily as a way of improving farm profits rather than being done with the intention of benefiting the animals (Ritter *et al.* 2019).

#### Technology usage

Participants often pointed to the benefits of using new technologies on pig farms. In the words of one participant: “Having the latest technology supports the animals and animal care, […] the environment, food safety and public health” [P4F6; veterinarian]. One participant described using technology in training employees to reduce language barriers:

“So, apps or technological programmes that you can develop that actually assist in training, and that […] opens up the world of reaching people that speak different languages … — Spanish is what I’m thinking — [this can] be used universally across the industry across the nation and brings more consistency” [P1F4; academic/scientist].

This view on training was supported by Rodriguez *et al.* (2018), who reported that mobile learning techniques that take into consideration aspects of culture, language and literacy were much more effective when delivering content on safety awareness to dairy farm workers compared to techniques that did not consider these aspects.

In line with the theme on antibiotic usage, some participants viewed technology as a way of tracking drug withdrawals in animals:

“… to track an animal that has been medicated all the way through the food supply, ensuring withdrawal times are being met and any tracking can be done. We’ve come a long way, but I think there’s also some animals that could slip through the cracks, [and] technology could help with that” [P3F4; industry].

Technology use in livestock production systems continues to increase (Neethirajan & Kemp 2021). However, adoption of technology is affected by several factors; Sun *et al.* (2021) identified performance expectancy, effort expectancy, social influence, personal innovation, and perceived risk as factors influencing pig farmers’ intention to adopt technology relating to food traceability. A survey of German citizens reported positive attitudes towards digital farming technology and increasing technology adoption rates by providing subsidies (Pfeiffer *et al.* 2021), suggesting that the use of these technologies on pig farms is unlikely to trigger public concern.

### Farm and industry culture

Changing farm and industry culture was discussed by participants. Specifically, discussions focused on shifting to animal welfare-centric farming, improving farmer welfare and support, increasing the appeal of pig farming for future generations, overcoming resistance to change, and training and education.

#### Animal welfare-centric farming

Participants described a shift towards management that was more considerate of animal needs; for example, “I’d like to see […] a way that we can enhance the animal’s experience…” [P3F6; academic/scientist]. Consideration that animals be provided a reasonably good life (even if some farm animals have a relatively short life) has received much attention in the literature (e.g. Yeates 2017; Stokes *et al.* 2020). This emphasis on a good life is driven in part by the value people place on attributes that resonate with their views on naturalness and positive emotional states (Sato *et al.* 2017) and suggests that ensuring a reasonably good life for the animals is important when discussing housing systems. Similarly, another participant commented: “Looking at the animal’s needs, [we need to be] more focused on what they need. It’s not from our perspective, but it’s from the pig’s perspective” [P7F2; industry]. A growing body of evidence suggesting that pigs are more likely to experience positive affective states (and less likely to experience negative states) when housed in more enriched environments compared to when to barren environments (Douglas *et al.* 2012; Mkwanazi *et al.* 2019).

#### Farmer welfare and support

Improving farmer and farm worker welfare was also desired by participants: “[…] worker well-being, mental health of the workers. I think that plays a lot into animal welfare. Make sure that [the workers] are well supported, hopefully better paid [and] better overall welfare at their jobs and like being there” [P6F1; academic/scientist]. Providing support to farmers during euthanasia decisions was also described as important to participants: “[…] being aware and proactive in dealing with caretaker fatigue and mental health issues” [P2F4; academic/scientist].

The focus on worker welfare on pig farms has to our knowledge received little attention (Jenkins & Perrow 1977), but this may increase in part due to the labour crisis experienced by many agriculture industries (Luo & Escalante 2017) and increased recognition of mental health issues in the farming community (for a review, see Younker & Radunovich 2022). Work by King *et al.* (2021) provides evidence linking poor cow welfare outcomes to reduced farmer mental health, suggesting this to be an important area worthy of more investigation.

#### Increasing the appeal of pig farming to future generations

Participants felt that pig farming needed to become more attractive in order to better attract a workforce. One participant grappled with the challenge of encouraging young people to work in agriculture:

“Some kids do want to go work in a barn, but a lot of them don’t and it’s just this huge labour issue. […] I don’t have the answer to how do you make it more glamorous? How do you make people want to go work with pigs? And then it comes back to do you need to have classes in high school where kids are exposed to ag? […] I don’t know how to plant the seed to make people want to work in pig farms” [P2F3; academic/scientist].

Succession planning within the family farm is a complex topic involving long-term planning by the farm owner and the prospective successor; decline in the willingness of children to take over the family farm has been identified as a critical area of global concern (Cavicchioli *et al.* 2018). Another challenge is the reduction in the number of people willing to work on farms more generally, potentially explained by low salaries; it is estimated that close to 40% of farm workers in the United States earn within 10% of the state-level minimum wage (Kandilov & Kandilov 2019).

#### Shifts in mindset

Participants called for a shift in thinking within the pig industry, specifically by becoming more proactive and decreasing resistance to change. For example, one participant commented on the industry’s way of dealing with consumer demands:

“…every industry goes through ups and downs of being very proactive and dealing with issues that come up from a consumer standpoint. [We] put it in the back of our minds… So … we [can’t be] resting on our laurels, and […] predicting the demands of the consumer as time goes on” [P1F4; academic/scientist].

Recognition that societal values are evolving, combined with a desire to address concerns, has been highlighted as important for the livestock sectors, particularly given the growing concern by citizens that production systems are overly exploitative of the animals (Boogaard *et al.* 2011). However, motivating changes in practice within the industry amongst farmers is not always easy. One participant discussed resistance to change:

“I think what’s important is once you get that information […] to the producers is [getting] them to understand…. I hear far too often, ‘This is how I’ve been doing things for 60 years’” [P5F1; academic/scientist].

Blackstock *et al.* (2010) described four different institutional mechanisms that could lead to farmer change: legal instruments; economic rewards; provision of advice; and voluntary collective actions. Within the EU, change has been largely driven by legislation and directives (EU Directive 98/58/EC 1998). Other jurisdictions have favoured voluntary actions, such as the industry-led Canadian Code of Practice for the Care and Handling of Pigs (NFACC 2014). However, whether industry-driven approaches are sufficient for the industry to stay aligned with evolving societal values remains to be seen and will likely depend in large part on how quickly industry is willing to address contentious issues. Regardless of mechanism, some change is likely inevitable, such that helping farmers adopt new practices should be a priority for the industry. The working experiences of the employees and the culture of the farm will influence the ease with which change is accomplished (Schneider *et al.* 1996). In the view of one participant, ensuring shared values on the farm was important to achieving change:

“I think the most important thing for me is that everybody on the farm has a universal understanding of the values that are associated with that farm. So, there’s not going to be anybody there that can say, I didn’t know” [P1F2; industry].

#### Training and education

Participants discussed how training and education of farming staff could be improved. In the words of one participant:

“I think that we need [to] tighten up on training. Not just orientation and then the first month or so that you have a new person on the facility but going back and following up with them in a month or two on the expectations and making sure that they don’t have any questions” [P8F2; industry].

Other work has emphasised the importance of training and attitudes of employees responsible for the day-to-day care in determining welfare on the farm (Losada-Espinosa *et al.* 2020). Training can also lead to broader behavioural and attitudinal changes among farm staff; for example, Ceballos *et al.* (2018) found that cattle handling training improved handlers’ attitudes and behaviours towards beef cattle, handling practices were better maintained over time, and that cattle performed fewer undesirable behaviours during handling.

### Farm-public interface

Participants discussed the relationship between farmers and the public, including farmer-public communication and education, changing public views of farming, and the participant’s own role in changing farm management practices.

#### Communication and education

Participants discussed increasing transparency between farms and the public, calling for “more transparency so that consumers can be better informed and make better choices. ‘Cause …all they have is the horrible undercover videos that they see on social media, or [the] sanitised picture that some industries are selling them” [P5F2; non-profit].

Educating the public was described as important: “Something to bring is more education just for the general public” [P4F5; industry]. The call by the industry participants to educate the public was not surprising as many have called for education of the public as a way of increasing acceptability of practices. In several studies completed by our group focused on understanding the views of dairy farmers (Ritter *et al.* 2020), cattle veterinarians (Sumner & von Keyserlingk 2018; Ventura *et al.* 2023), and animal science students (Ritter *et al.* 2021), participants called for similar efforts to educate the public, with the belief that this would improve public acceptance of current practices. However, scholarly work on this topic suggests that educational efforts are not always successful in shifting public attitudes in the direction desired (e.g. Ventura *et al.* 2016; Hötzel *et al.* 2017). One-way information reflecting farmer values will likely fail to improve public acceptance, particularly when trust has already been lost (Arnot *et al.* 2016). Other approaches, such as facilitating shared learning experiences, are likely to have more success (Benard & de Cock Buning 2013).

#### The public’s role in farming practices

Participants discussed improving public views of farming. One participant called for increasing transparency as it would eliminate:

“[…] any social stigma associated with animal production or working in animal agriculture. And that has to come hand-in-hand with there no longer [being] anything to hide or anything to be ashamed of for anyone working in this field” [P5F2; non-profit].

Another participant felt that the public should have less of a role in influencing farm practices:

“I would hope in the future that consumers don’t gain the power to dictate how producers raise their pigs. We need to do the research behind what is best for the welfare of the pig and for production in order to provide that protein source to people, but at the same time, meeting the needs of the pig and not necessarily just doing something like giving them beach balls because the public decides that pigs need beach balls” [P4F1; industry].

Considering that public concern for farm animal welfare is increasing (Alonso *et al.* 2020), it seems unlikely excluding the public voice from such discussions will be socially sustainable. Indeed, a recent study by Regan and Kenny (2022) found that members of the public expressed a desire for increased and two-way engagement with farmers.

### Sustainability

A sustainable system is one that is economically viable, environmentally friendly and socially acceptable, but the latter pillar is the most ill-defined and often challenging for animal agriculture (Arvidsson Segerkvist *et al.* 2020). Thus, it was not surprising that the participants in the current study focused their discussion on the economic and environmental pillars of sustainability.

#### Economic sustainability

Participants described the need to ensure profitability for pig farmers, for example: “One of the things I want to make sure … is that it has to be equitable for the producer long-term. They have to make a profit. That’s what they’re in the business for” [P3F4; industry]. Another participant expressed apprehension regarding losing farmers due to lack of income, drawing from their experiences in the dairy industry:

“I have friends whose families have dairy, and it’s just so hard for them to make money… I don’t want the swine industry to get to that point, but if there’s no open communication between anyone, I think it might eventually get there, and that scares the crap out of me” [P4F3; industry].

One challenge will be to balance economic viability in the near term with the ability to pivot in response to market changes, including responding to new pressures from retailers and other actors along the value chain (Christensen *et al.* 2019; Esbjerg *et al.* 2022). Pressures that are frequently imposed in response to issues, like animal welfare that arguably fall into the social dimension of sustainability. For instance, due to concerns regarding hen welfare, regulatory and market initiatives have resulted in a shift away from housing hens in battery cages to alternative housing systems (Scrinis *et al.* 2017). Similarly, Marchant-Forde and Boyle’s (2020) analysis of the early months of the COVID pandemic highlighted the fragility of intensive industries dependent on high throughput of animals in the processing chain. The pig industry must be prepared to meet intersecting challenges to animal, environmental and worker welfare.

#### Environmental sustainability

Participants discussed ways in which future pig farms could become more environmentally sustainable. One participant discussed this in terms of waste management, drawing connections between environmental sustainability and animal and human well-being:

“I think one aspect of this ideal farm is that waste management systems have to be significantly improved … [this will] improve the welfare of the workers, the animals, the environment …. and hopefully, those waste products are recirculated or reutilised in some way that feeds back into the system” [P5F2; non-profit].

Another participant offered: “[…] making sure pig farms are as sustainable as possible, managing their waste properly…” [P6F1; academic/scientist].

Some research shows that public awareness of the environmental impact of food production is low (MacDiarmid *et al.* 2016; Dopelt *et al.* 2019), but other work suggests that farmer and public attitudes towards agriculture-related conservation issues displayed similar levels of concern regarding the environment (Howley *et al.* 2014).

### Limitations

This study is based upon a convenience sample of participants who were attending a conference on pig welfare and who were willing to participate in our focus groups sessions. Our findings are thus not intended to be generalisable to all participants attending the conference, nor the broader pig industry either in North America or any other region. Future studies should include stakeholders from across the value chain and their decision-making processes involved in the care and handling of pigs. Our focus groups were also made up of a mixture of actors along the value chain, including farmers, veterinarians and scientists amongst others; we encourage future work to also focus on each of these groups independently.

### General discussion

This study sought to describe the views of individuals affiliated with the pig industry in relation to their vision for the future of pig farming. Although the issues discussed varied among focus groups, participants largely focused on challenges encountered when working on pig farms in the present, such as minimising disease, improving health, euthanasia practices and employee training. Participants believed that technology has and will continue to play a role in the future, particularly for early disease detection.

Despite being prompted to discuss the ‘must haves’ envisioned for the industry, and the steps needed to implement this vision, the focus group participants spent the majority of time focusing on their current challenges. Ritter *et al.* (2019) reported a similar focus in their work with Canadian dairy farmers.

Participants discussed factors they believed were required for farms to remain economically viable and compliant with environmental regulations. Although they did not raise issues pertaining to the social pillar of sustainability, they did discuss the role of the public. While some participants called for the need to evaluate the social acceptability of existing practices and encouraged transparency between the industry and the public, others expressed frustration with the public, arguing that they should have less influence on practices. Future research should consider adopting participatory methods where all stakeholders along the value chain, including the public, are included from the outset in discussions identifying what characteristics are associated with a sustainable pig industry (for a review, see Bolton & von Keyserlingk 2021). Given the focus of the symposium, participants in the current study were likely more aware of animal health and welfare issues than would be typical of others working in the pig industry. Moreover, our findings are based on a convenience sample of participants from a single conference; as such, the results should not be seen as generalisable to the US pig industry as a whole. We encourage future work to document the views of a broader spectrum of this industry, and to assess the views on those working with pigs in other parts of the world. We remind readers that there are cultural differences with respect to the timelines applied when assessing the impact of change on the viability of an industry; in North America some businesses may have a shorter term focus relative to those in some other regions including Europe (Haga *et al.* 2019).

## Animal welfare implications and conclusion

To our knowledge, this is the first study to describe views from those working within or adjacent to the US pig industry on the need for change in how pigs are cared for. We found that perceived ‘must haves’ of good animal care were rarely discussed alone but rather in conjunction with current challenges of pig farming, such as employee management and economic factors. Participants were often less focused on societal concerns, potentially increasing the risk of a disconnect between public expectations practices within the pig industry. Continued research in this area is needed given the influence these stakeholders can have on the welfare of pigs on farms.
